# Single Administration of Tripeptide α-MSH(11–13) Attenuates Brain Damage by Reduced Inflammation and Apoptosis after Experimental Traumatic Brain Injury in Mice

**DOI:** 10.1371/journal.pone.0071056

**Published:** 2013-08-05

**Authors:** Eva-Verena Schaible, Arne Steinsträßer, Antje Jahn-Eimermacher, Clara Luh, Anne Sebastiani, Frida Kornes, Dana Pieter, Michael K. Schäfer, Kristin Engelhard, Serge C. Thal

**Affiliations:** 1 Department of Anesthesiology, Medical Center of the Johannes Gutenberg-University Mainz, Mainz, Germany; 2 Focus Program Translational Neuroscience, Medical Center of the Johannes Gutenberg-University Mainz, Mainz, Germany; 3 Institute of Medical Biostatistics, Epidemiology and Informatics, Johannes Gutenberg-University Mainz, Mainz, Germany; Univ. Kentucky, United States of America

## Abstract

Following traumatic brain injury (TBI) neuroinflammatory processes promote neuronal cell loss. Alpha-melanocyte-stimulating hormone (α-MSH) is a neuropeptide with immunomodulatory properties, which may offer neuroprotection. Due to short half-life and pigmentary side-effects of α-MSH, the C-terminal tripeptide α-MSH(11–13) may be an anti-inflammatory alternative. The present study investigated the mRNA concentrations of the precursor hormone proopiomelanocortin (POMC) and of melanocortin receptors 1 and 4 (MC1R/MC4R) in naive mice and 15 min, 6, 12, 24, and 48 h after controlled cortical impact (CCI). Regulation of POMC and MC4R expression did not change after trauma, while MC1R levels increased over time with a 3-fold maximum at 12 h compared to naive brain tissue. The effect of α-MSH(11–13) on secondary lesion volume determined in cresyl violet stained sections (intraperitoneal injection 30 min after insult of 1 mg/kg α-MSH(11–13) or 0.9% NaCl) showed a considerable smaller trauma in α-MSH(11–13) injected mice. The expression of the inflammatory markers TNF-α and IL-1β as well as the total amount of Iba-1 positive cells were not reduced. However, cell branch counting of Iba-1 positive cells revealed a reduced activation of microglia. Furthermore, tripeptide injection reduced neuronal apoptosis analyzed by cleaved caspase-3 and NeuN staining. Based on the results single α-MSH(11–13) administration offers a promising neuroprotective property by modulation of inflammation and prevention of apoptosis after traumatic brain injury.

## Introduction

Alpha-melanocyte stimulating hormone (α-MSH), a tridecapeptide originated from proopiomelanocortin (POMC), is a neuropeptide with immunomodulatory properties. It acts as a suppressor of proinflammatory cytokine production e.g. interferon-γ (IFN-γ), tumor necrosis factor-α (TNF-α), interleukin-1 (IL-1), IL-6, and IL-8 [1]–[6]. Additionally, it induces IL-10 expression, which is one of the most important mediators of the anti-inflammatory effect of α-MSH [7]. However, the potential therapeutic value of α-MSH is limited by its short half-life and melanotropic effects. Thus many investigations were performed to identify related peptides without pigmentary action, but similar immunomodulatory properties.

The C-terminal tripeptide α-MSH(11–13), also known as KPV (_L_-Lys-_L_-Pro-_L_-Val), was found to exhibit anti-inflammation without induction of cutaneous pigmentation in a frog skin bioassay [8]. The underlying molecular mechanisms of the α-MSH(11–13) anti-inflammatory properties have not been determined in detail, but are related to an inhibition of TNF-α, IL-6, and nitric oxide production in a model of LPS-stimulated murine microglial cell culture [9]. The literature on tripeptide interaction with melanocortin receptor subtype 1 (MC1R) remains controversial. In vitro and in vivo studies failed to show an interaction of α-MSH(11–13) with MC1R and rather suggested, due to structural similarity, an effect by IL-1β mediated activation of IL-1 receptors that enhances anti-inflammation by anti-migratory effects of neutrophils [10]. In contrast, the tripeptide induced dependent on the presence of MC1R a calcium signal in MC1R-transfected chinese hamster ovary (CHO)-K1 cells that led to inhibition of TNF-α-stimulated activation of nuclear factor (NF)-κB transcription factor [11]. Furthermore, it has been recently shown, that α-MSH(11–13) not only reduces activation of NF-κB, but also decreases its translocation into the nucleus by interaction with importin family proteins [12]. Likewise an interaction with MC4R, the common melanocortin receptor in the brain [13], is unclear. However, MC4R seems to have neuroprotective properties, such as a selective stimulation of MC4R after transient global cerebral ischemia in gerbils was associated with less hippocampal inflammation and apoptosis [14].

The aim of the present study was to characterize posttraumatic mRNA expression of POMC, MC1R, and MC4R and to investigate the effect of α-MSH(11–13) on secondary lesion volume, posttraumatic inflammation, microglial activity, and apoptosis after experimental traumatic brain injury (TBI) in mice.

## Methods

### Animals

Altogether 96 two months old male C57Bl/6N mice (Charles River Laboratory, Sulzfeld, Germany; 21–35 g) were investigated. All animal procedures were performed in compliance with institutional guidelines of the Johannes Gutenberg-University Mainz, Germany. All experiments were approved by the Animal Ethics Committee of the Landesuntersuchungsamt Rheinland-Pfalz (protocol numbers 23177-07/G 11-1-006 and 23177-07/G 10-1-028).

### Experimental Groups

A) The total animal number in the analysis of time-dependent mRNA expression after TBI was 60. A time-course of POMC, MC1R, MC4R, and cyclophilin A (PPIA, housekeeping gene) mRNA expression was determined in brain tissue from healthy animals (naive) and in pericontusional brain tissue 15 min, 6, 12, 24 and 48 h after controlled cortical impact injury (CCI, n = 10 each).

B) The second part of the study, which investigated the impact of α-MSH(11–13) injection after TBI, compassed a total of 36 mice. The effect of α-MSH(11–13) on secondary histopathological brain damage, neuroinflammation, and apoptosis was determined in animals randomized to intraperitoneal injection of vehicle (0.9% NaCl) or α-MSH(11–13) (1 mg/kg) 30 min post CCI (n = 10 each). The primary lesion was quantified in a separate set of animals 15 min after injury (n = 6). A sham group underwent all procedures with exception of the CCI (intraperitoneal injection of 0.9% NaCl 30 min post CCI, n = 10).

### Drug Preparation

Prior to use α-MSH(11–13) (Bachem AG, Bubendorf, Switzerland) was dissolved in normal saline (0.9% NaCl). After randomization vehicle solution (0.9% NaCl) or α-MSH(11–13) was injected intraperitoneally 30 min after trauma by an investigator blinded towards group allocation.

### Traumatic Brain Injury

Traumatic brain injury was performed as described previously [15], [16]. Mice were anesthetized with isoflurane (Abbott, Wiesbaden, Germany) mixed with 40% O_2_ and 60% N_2_ (induction in a bell jar filled with 4% isoflurane, maintenance via face mask with 1.6% isoflurane) and placed in a stereotactic apparatus (Kopf Instruments, Tujunga, USA). A feedback controlled heating pad (Hugo Sachs, March-Hugstetten, Germany) maintained rectal measured body temperature at 36.5°C. After midline incision of the skin a 4×4 mm craniotomy using a high-speed drill (Paggen, Starnberg, Germany) was carried out over the right parietal cortex between the sagittal, lambdoid and coronal sutures. A pneumatic brain trauma was performed on the dura-covered brain via CCI. The custom fabricated impactor (L. Kopacz, Mainz, Germany) was used with a 3 mm diameter of impactor tip, an impact velocity of 8 m/s, an impact duration of 150 ms and 1.5 mm brain penetration. After injury the initially removed cranium was flapped back immediately and fixed with conventional tissue glue Histoacryl (B. Braun Melsungen AG, Melsungen, Germany). The suture of the skin incision finished the surgery. Mice were placed for 2 h in an incubator heated to 33°C and at a humidity of 35% (IC8000, Draeger, Lübeck, Germany) to recover in their individual cages.

### Neurological Function

Body weight and a neurological severity score (NSS) were determined before and 23 h after CCI by an investigator blinded towards group allocation. A modified 15-point neuroscore was applied to evaluate the functional outcome [17]. General behavior, alertness, motor ability, and balancing were rated by 10 different tasks. Depending on the performance each task was rated as zero (normal) or with up to three points (failed task, **Table 1**). One day prior to the experiments, all animals were tested with the NSS. Healthy animals were required to pass the test with 0 or 1 point to be enrolled in the study. NSS of each mouse was calculated by subtraction of post-CCI values with pre-CCI data. Severely impaired mice received up to 15 points.

**Table 1:** Criteria of the Neurological Severity Score (NSS).

Task	Points
Presence of mono- or hemiparesis	1
Inability to walk on a 3 cm-wide beam or walking with one-time foot displacement	2 or 1
Inability to walk on a 2 cm-wide beam or walking with one-time foot displacement	2 or 1
Inability to walk on a 1 cm-wide beam or walking with one-time foot displacement	2 or 1
Inability to balance on a round stick (0.5 cm diameter)	1
Inability to balance on a square stick (0.4×0.4 cm)	1
Inability to walk straight line	1
Failure to exit from circle (30 cm diameter) within 2 min or 1 min or 30 sec	3 or 2 or 1
Loss of startle behavior	1
Loss of seeking behavior	1
**Total Maximum**	**15**

### Histological Evaluation

The brains were carefully removed from isoflurane-anesthetized animals, immediately frozen in powdered dry ice and stored at −20°C. Brains were cut in coronal plane with a cryostat (HM 560 Cryo-Star, Thermo Fisher Scientific, Walldorf, Germany). The first slide was taken at bregma +3.14 mm according to the Mouse Brain Library atlas (http://www.mbl.org). 10 µm thick sections were collected every 500 µm, placed on Superfrost Plus slides (Thermo Fisher Scientific) and stained with cresyl violet. The area of both hemispheres and region of contused brain tissue, defined as region with lack of cresyl violet staining, were analyzed using a computerized image system (Optimas 6.51, Optimas Corporation, Bothell, USA) by an investigator blinded to experimental groups. Contusion volume was calculated by multiplying contusion areas obtained from 16 consecutive sections with a 500 µm distance between histological sections based on following formula: 0.5*(A1+ A2+ …+An).

### Gene Expression Analysis

During histologic processing tissue samples of the contused brain region were sampled from frozen brains. 40 coronal trimming slices (30 µm) at the level of contusion were separated between the left and right hemisphere and then bisected into an upper and lower quadrat. The upper right quadrants containing the contused brain tissue were transferred into a pre-cooled tube and frozen in liquid nitrogen prior to long-term storage at −80°C. The mRNA quantification by real-time quantitative polymerase chain reaction (qPCR) was performed as previously described in detail [18]. Same amounts of cDNA were utilized in duplicates and amplified with Maxima Probe qPCR Master Mix (Fermentas GmbH, St. Leon-Rot, Germany) for POMC and MC4R, Kapa Probe Fast qPCR (peqlab biotechnology GmbH, Erlangen, Germany) for MC1R, LightCycler® 480 Probes Master (Roche, Grenzach-Wyhlen, Germany) for IL-1β, ABsolute™ Fast QPCR Mix (Thermo Scientific) for cyclophilin A or ABsolute™ Blue QPCR SYBR Green Mix (Thermo Scientific) for TNF-α. The absolute copy numbers of the target genes were normalized against the absolute copy numbers of cyclophilin A (PPIA) as a housekeeping gene [15]. Applied primers and probes are listed in **Table 2**.

**Table 2:** Primer and Probes with optimized temperature conditions for real-time PCR.

Polymerase Chain Reaction Assay	Oligonucleotide Sequence (5′–3′)	GeneBank No.
**[amplicon size, annealing temperature, A, E]**		
**Cyclophilin A (PPIA)**	Forward: 5′-GCGTCTSCTTCGAGCTGTT-3′	NM_008907
[146 bp, 55°C, A: 10 s, E: 15 s]	Reverse: 5′-RAAGTCACCACCCTGGCA-3′	
	Cy5: Cy5-TTGGCTATAAGGGTTCCTCCTTTCACAG-Phos	
	FL: 5′-GCTCTGAGCACTGGRGAGAAAGGA-FL	
**POMC**	Forward: 5′-TAGAGTTCAAGAGGGAGCTG-3′	NM_008895.3
[345 bp, 55°C, A: 30 s, E: 30 s]	Reverse: 5′- CCGACTGTGAAATCTGAAAGG-3′	
	Cy5: Cy5-ATGACCTCCGAGAAGAGCCAGA-Phos	
	FL: 5′-AAGGACAAGCGTTACGGTGGCT-FL	
**MC1R**	Forward: 5′-CCATGAAAACCCAGCCATAG-3′	NM_008559.2
[371 bp, 55°C, A: 10 s, E: 15 s]	Reverse: 5′-TCCTCTCACCCAAACTTCAC-3′	
	Cy5: Cy5-AGCCTCCTTGCCATCTTCCCTA-Phos	
	FL: 5′-CAGTTGAAATGCTAAGGTCAGAGGGA-FL	
**MC4R**	Forward: 5′-TGAGCATTCAGAAGCACCAG-3′	NM_016977.3
[397 bp, 55°C, A: 30 s, E: 30 s]	Reverse: 5′-CACCCAGAGTCACAAACACC-3′	
	Cy5: Cy5-TCCTTTGCGAGTTCCGCTGCTT-Phos	
	FL: 5′-TGAGCCGAACCCAGAAGAGACCAACAA-FL	
**TNF-alpha**	Forward: 5′-TCTCATCAGTTCTATGGCCC-3′	NM_013693
[212 bp, 62°C, A: 10 s, E: 10 s]	Reverse: 5′-GGGAGTAGACAAGGTACAAC-3′	
**IL-1 beta**	Forward: 5′-GTGCTGTCGGACCCATATGAG-3′	NM_008361
[348 bp, 55°C, A: 10 s, E: 15 s]	Reverse: 5′-CAGGAAGACAGGCTTGTGCTC-3′	
	Cy5: Cy5-CAGCTGGAGAGTGTGGATCCCAAGC-Phos	
	FL: 5′-TAATGAAAGACGGCACACCCACCC-FL	

Forward, sense primer; Reverse, antisense primer; Cy5, Cyanine 5; Phos, Phosphate; FL, fluorescein; A, annealing time; E, extension time.

### Ionized Calcium Binding Adaptor Molecule-1 (Iba-1) Staining

For immunohistochemical staining of activated microglia cryosections were fixed in 4% paraformaldehyde, rinsed with PBST, phosphate-buffered saline (PBS) with 0.3% Triton× (Sigma, St. Louis, USA), and incubated in blocking solution consisting of PBS with 5% normal goat serum (DAKO, Glostrup, Denmark). The antibodies were solved in PBST. At room temperature sections were incubated over night with rabbit anti-Iba-1 antibody (1∶1500; WAKO Pure Chemical Industries, Osaka, Japan). After rinsing biotinylated anti-rabbit IgG (H+L) (Vector Laboratories Inc., Burlingame, USA) was applied as secondary antibody. A peroxidase, ABC-Complex (Vector Laboratories Inc., Burlingame, USA), was applied after rinsing. The non-bound complex was washed out and then the chromogen DAB (DAKO, Glostrup, Denmark) was added. Afterwards, a background staining with hemalum solution (AppliChem GmbH, Darmstadt, Germany) was performed. Total amount of positive cells was counted in ipsi- and contralateral cortex (region of interest: 0.52 mm×0.65 mm) at bregma −2.36 mm according to Mouse Brain Library atlas (http://www.mbl.org) by an investigator blinded to group allocation. In addition, Iba-1 positive cells were distinguished by their number of branches (0 up to 6 branches).

Upon activation, microglia retracts and thickens its branches when transforming in a reactive form [19]. Therefore, reduction of branches reflects activation of the cells. The ratio of branched cells over the total number of cells (in percent) was determined in the ipsi- and contralateral hemisphere with categorization into three groups: 0–1 branch, 2 branches and >2 branches.

### Neuronal Expression of Activated Caspase-3

For double immunofluorescence staining of cleaved caspase-3 and neuronal nuclei (NeuN) cryosections were fixed in 4% paraformaldehyde, rinsed with PBS and incubated in blocking solution containing 0.1% Triton X 100. Sections were incubated at 4°C over night with anti-active caspase-3 (1∶100; rabbit, BD Pharmingen, Franklin Lakes, US) and anti-NeuN (1∶500; mouse, Merck Millipore, Darmstadt, Germany). After several washes in PBS, Alexa Fluor 568 goat anti-mouse IgG (H+L) and Alexa Fluor 488 goat anti-rabbit IgG (H+L) were applied (1∶300 and 1∶200; both purchased from Invitrogen Life Technologies, Darmstadt, Germany). Total number of double positive cells was counted in gyrus dentatus at bregma −2.36 mm according to Mouse Brain Library atlas (http://www.mbl.org) by an investigator blinded to group allocation.

### Statistics

Statistical analysis was performed using Sigma Plot 11 Statistical Software package (Systat Software, Inc., USA). Exact Wilcoxon-Mann-Whitney tests were used in each study for pairwise comparisons and for each outcome adjustments for multiple testing using Bonferroni-Holm were done. As this is an explorative study p-values are given for descriptive reasons only. Results are presented as mean ± standard deviation (S.D.).

## Results

### Mortality

All animals of primary lesion, sham and vehicle group survived the observation period. In α-MSH(11–13) injected group one animal was euthanized before injection of treatment because of persistent seizures after injury.

### Time Course of POMC, MC1R, and MC4R mRNA Expression

**Figure 1:**
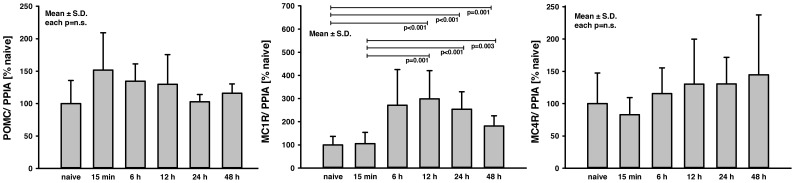
Time response of POMC, MC1R and MC4R mRNA expression. The mRNA expression of POMC, MC1R and MC4R was measured in contused brain tissue by real-time RT-PCR (n = 10 per group). POMC and MC4R were only slightly changed at any time after trauma (p = n.s.). MC1R increased over time and was elevated 3-fold at 12 h (p<0.001), 2.5-fold at 24 h (p<0.001) and 1.8-fold at 48 h (p = 0.001) after brain trauma compared to naive brain tissue. Similar increases were measured at 12 h (2.8-fold; p = 0.001), 24 h (2.4-fold; p<0.001) and 48 h (2-fold; p = 0.003) compared to 15 min. Data were analyzed using exact Wilcoxon-Mann-Whitney test and adjusted for multiple comparisons using Bonferroni-Holm. As this is an explorative study p-values are given for descriptive reasons only. All bar charts show mean ± S.D.

All melanocortin peptides were separated from the precursor POMC. To specify the time-dependent regulation of POMC as well as in the brain prevalent melanocortin receptors MC1R and MC4R, samples from naive and contused brain tissue were quantified at different time points. POMC and MC4R mRNA expression was only slightly changed after trauma compared to naive (each p = n.s.). MC1R mRNA expression increased over time with a 3-fold peak at 12 h and returned to 2.5-fold at 24 h and 1.8-fold at 48 h compared to naive (12 h: p<0.001; 24 h: p<0.001; 48 h: p = 0.001; **Figure 1**).

### Neurological Outcome

**Figure 2:**
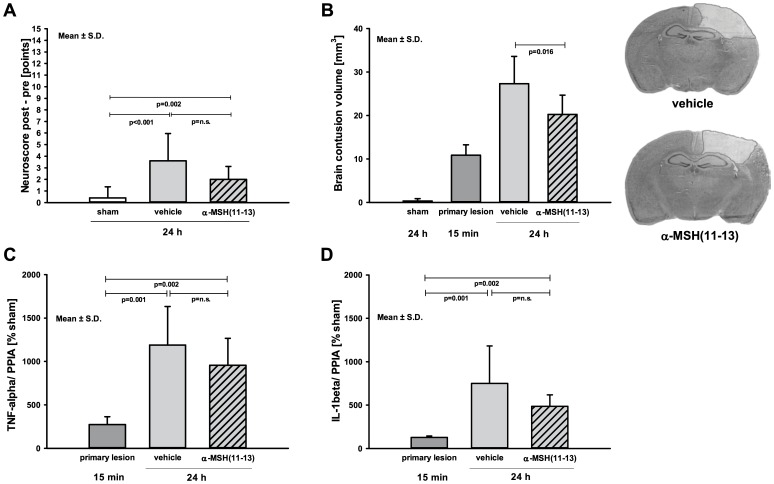
Effect of α-MSH(11–13) on secondary brain injury. (**A**) Neurological function was evaluated by neurological severity score (NSS; 0 point = no impairment; 15 points = maximal impairment; **Table 2**). NSS of each mouse is the difference of NSS post- to pre-CCI. 23 h after modest brain trauma all mice presented a moderate impaired neurological function with no difference between treatment groups (α-MSH(11–13) vs. vehicle [points]: 2±1.12 vs. 3.6±2.34). (**B**) Contusion volume increased over time between 15 min (primary lesion) and 24 h after experimental brain trauma (CCI). Tripeptide α-MSH(11–13) administrated mice developed an obvious smaller lesion volume compared to vehicle analyzed in cresyl violet stained brain slices (α-MSH(11–13) vs. vehicle [mm^3^]: 21±3.39 vs. 27.67±4.81; p = 0.016). The mRNA expression of inflammatory marker genes was determined in contused brain tissue by real-time RT-PCR. (**C**) TNF-α and (**D**) IL-1β were upregulated after trauma. TNF-α [% sham] mRNA expression showed a 4-fold increase from primary lesion to vehicle group (p = 0.001) and a 3.5-fold increase in tripeptide-injected group (p = 0.002). The mRNA expression of IL-1β was about 6-fold increased in vehicle (p = 0.001) and 4-fold increased in α-MSH(11–13) group (p = 0.002) at 24 h after injury. Both inflammatory marker genes showed no relevant different expression between treatment groups (p = n.s.). Data were analyzed using exact Wilcoxon-Mann-Whitney test and adjusted for multiple comparisons using Bonferroni-Holm. As this is an explorative study p-values are given for descriptive reasons only. All bar charts show mean ± S.D.

Consistent with modest brain trauma induction all animals demonstrated a moderate impaired neurological function at 23 h after trauma compared to sham. There was no relevant difference between treatment groups (α-MSH(11–13): 2±1.1 points; vehicle: 3.6±2.3 points; p = 0.139; **Figure 2** **A**).

### Effect of α-MSH(11–13) Fragment on Secondary Brain Damage

To determine whether a single administration of the tripeptide acetate salt of α-MSH is neuroprotective, the tripeptide was administered 30 min post CCI. As expected, brain lesion volume increased over time between 15 min (primary lesion) and 24 h. After the observation period, the tripeptide injected animals developed a considerable smaller brain lesion (-24%) compared to vehicle as examined in cresyl violet stained brain slices (α-MSH(11–13): 21.0±3.4 mm^3^; vehicle: 27.7±4.8 mm^3^; p = 0.016; **Figure 2** **B**).

### Influence of α-MSH(11–13) on Inflammatory Marker Gene Expression after Brain Trauma

The inflammatory marker genes TNF-α and IL-1β were upregulated over time from 15 min to 24 h after trauma. The expression levels were by trend smaller in α-MSH(11–13) group for both TNF-α (α-MSH(11–13): 3.5-fold; vehicle: 4-fold; p>0.069; **Figure 2** **C**) and IL-1β (α-MSH(11-13): 4-fold; vehicle: 6-fold; p = 0.158; **Figure 2** **D**) compared to vehicle treated animals.

### Iba-1 Activity After α-MSH(11–13) Administration

**Figure 3:**
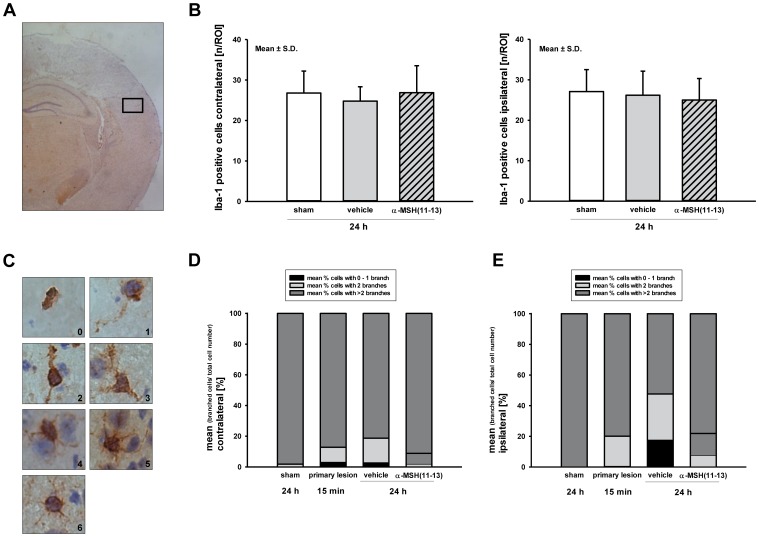
Effect of α-MSH(11-13) on microglial activity. As a marker for cerebral inflammation microglial cells were quantified by staining for Iba-1. (**A+B**) Total amount of positive cells was counted in an ipsilateral and contralateral predefined window with no relevant difference between groups (ipsilateral: α-MSH(11-13) 25.44±4.85 cells/mm^3^ vs. vehicle 27.9±6.28 cells/mm^3^; contralateral: α-MSH(11-13) 25.44±4.26 cells/mm^3^ vs. vehicle 25.5±4.88 cells/mm^3^; each p = n.s.). (**C**) In series Iba-1 positive cells were categorized in cells with 0 up to 6 branches. (**C**) The ratio of branched cells to total cell number in contralateral brain tissue is nearly adapted between treatment groups. (**E**) Counting of branches of ipsilateral tissue categorized into 0–1, 2, and >2 branches and summarized as mean (number of branched cells/total cell number [%]) illustrated a less activated status of microglia after α-MSH(11-13) administration compared to vehicle. Numeric data are shown in **Table 3**. Considering cells with one or no branch as activated, there was a lesser degree of activation in the α-MSH(11-13) treated group indicating a reduced inflammatory state (α-MSH(11-13) 7.78±8.76% vs. vehicle 17.87±9.65%, p = 0.033). Relating to cells with a maximum of two branches as activated cells, the difference was even more evident (α-MSH(11-13) 21.92±13.05% vs. vehicle 47.65±14.1%, p = 0.002; numeric data presented as mean ± S.D. are shown in **Table 4**). As this is an explorative study p-values are given for descriptive reasons only.

As additional marker for cerebral inflammation microglial cells were quantified by Iba-1 staining (**Figure 3** **A**). Total amount of positive cells was counted in ipsilateral and contralateral hemisphere and showed no obvious difference between groups (ipsilateral: α-MSH(11-13) 25.4±4.9 cells/mm^3^ vs. vehicle 27.9±6.3 cells/mm^3^; p = 0.711; contralateral: α-MSH(11-13) 25.4±4.3 cells/mm^3^ vs. vehicle 25.5±4.9 cells/mm^3^; p = 0.234; **Figure 3** **B**). The numbers of branches of microglia decrease upon activation. Therefore the numbers of branches of Iba-1 positive cells were counted (**Figure 3** **C**), categorized to 0–1, 2 and >2, and presented as % of total Iba-1 positive cells (**Table 3**). Sham operated animals showed more than two branches on average in 99% of Iba-1 positive cells. In the healthy contralateral hemisphere on average 80-90% of Iba-1 cells demonstrated more than two branches with only slight differences between groups (**Figure 3** **D**). Microglia in the traumatized, ipsilateral hemisphere of α-MSH(11-13) treated animals showed more ramified and less branch-rarefied cells compared to vehicle, indicating a lower grade of activation (mean of cells with 0–1 branch: α-MSH(11-13) 7.78±8.76% vs. vehicle 17.87±9.65%; p = 0.033; mean of cells with 0–2 branches: α-MSH(11-13) 21.92±13.05% vs. vehicle 47.65±14.1%; p = 0.002; **Figure 3** **E,** **Table 4**).

**Table 3:** Mean percentage (%) of branched cells to total cells of ipsilateral and contralateral side after Iba-1 staining.

ipsilateral	mean % cells with 0–1 branch	mean % cells with 2 branches	mean % cells with >2 branches
sham	0	0.303	99.697
primary lesion	0.725	19.381	79.894
vehicle	17.867	29.775	52.358
α-MSH(11-13)	7.781	14.137	78.082
**contralateral**			
sham	0	1.852	98.148
primary lesion	3.39	9.449	87.161
vehicle	3.077	15.691	81.232
α-MSH(11-13)	1.986	6.916	91.098

**Table 4:** Comparison of mean percentage (%) of ipsilateral Iba-1 positive cells with ≤1 or 2 branches to total cell number.

number of branches	α-MSH(11-13)	vehicle	p-value
	[mean % cells ± S.D.]	[mean % cells ± S.D.]	
0–1	7.78±8.76	17.87±9.65	p = 0.033
0–2	21.92±13.05	47.65±14.1	p = 0.002

### Effect of α-MSH(11-13) on Apoptosis

**Figure 4:**
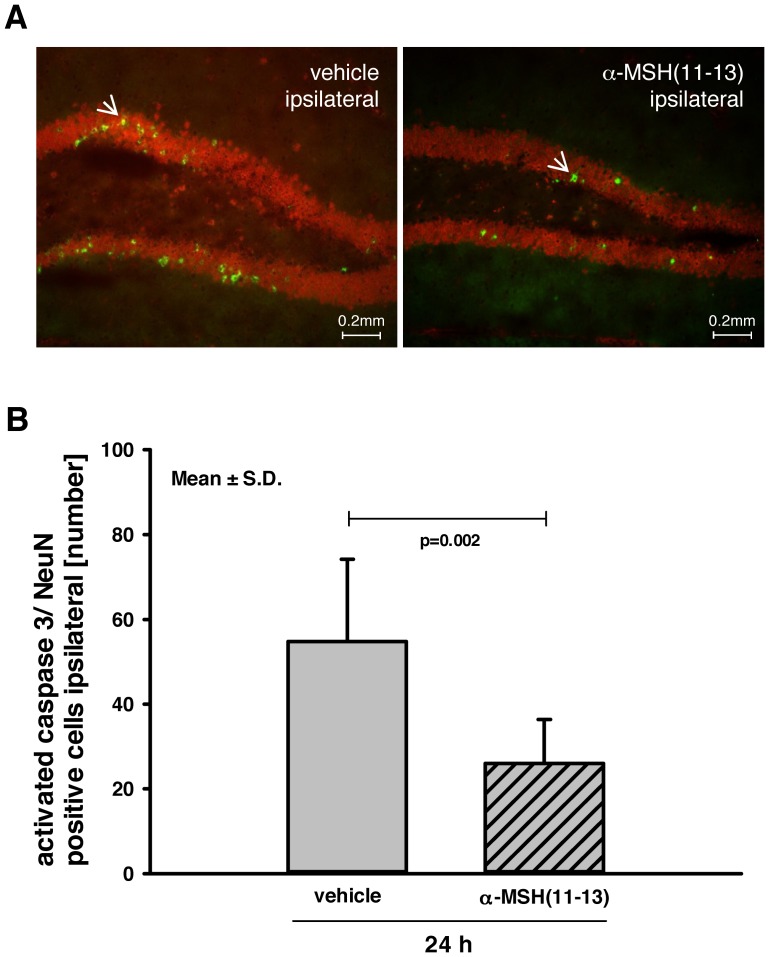
Effect of α-MSH(11-13) on apoptosis. (**A**) To determine the influence of α-MSH(11-13) on apoptosis, analysis of apoptotic cells was performed using immunofluorescent double-staining of cleaved caspase-3 (green) and neuronal nuclei (red). White arrows point to apoptotic cells. (**B**) In the quantitative analysis, α-MSH(11–13) administered group showed obvious less apoptotic cells compared to vehicle (each n = 9; one slice of vehicle was excluded due to damage; 26±10.34 vs. 54.78±19.42; p = 0.002). Data were analyzed using exact Wilcoxon-Mann-Whitney test and adjusted for multiple comparisons using Bonferroni-Holm. As this is an explorative study p-values are given for descriptive reasons only. All bar charts show mean ± S.D.

To determine the influence of α-MSH(11-13) on neuronal apoptosis, immunofluorescent double-staining for cleaved caspase-3 (green) and NeuN (red) was performed to visualize the number of apoptotic neurons (**Figure 4** **A**; white arrow points to apoptotic cell). The α-MSH(11-13) administered group showed less double positive cells (26±10.3 cells/ROI) compared to vehicle (54.8±19.4 cells/ROI; p = 0.002; **Figure 4** **B**) indicating reduced neuronal apoptosis in the treatment group.

## Discussion

The present data demonstrate for the first time a neuroprotective effect of the α-MSH tripeptide fragment (11–13) in an experimental TBI model. A single injection of α-MSH(11–13) diminishes secondary brain lesion expansion possibly by reducing apoptotic cell damage. Despite the well described anti-inflammatory effects of α-MSH(11–13) it did not influence the posttraumatic gene expression of TNF-α and IL-1β as well as the total amount of Iba-1 positive cells. The degree of activation of Iba-1 positive cells in the ipsilateral hemisphere was distinctly reduced by the tripeptide. These data suggest that neuroprotection by α-MSH(11–13) might be mediated not exclusively by its immunomodulatory action but also by its anti-apoptotic effect.

Due to the beneficial modulating effects of α-MSH and its derivatives on inflammation, infectious processes, or energy homeostasis, these proteins have attracted attention of many investigators [20]–[23]. Decreased α-MSH plasma levels were associated with poor neurologic outcome in a rat model of middle cerebral artery occlusion and also in patients with ischemic stroke [24], [25]. Also in humans, α-MSH concentrations were lessened in critically injured trauma patients as well as after subarachnoid hemorrhage or TBI [26], [27].

To understand the role of α-MSH after brain injury, the first part of the study characterized the trauma-induced changes of proteins associated with the production and function of α-MSH. Therefore, the influence of TBI on the cerebral regulation of the precursor hormone POMC, of which α-MSH is endogenously derived, as well as the melanocortin receptors MC1R and MC4R, which orchestrate the pleiotropic effects of the melanocortin peptides, were investigated. In the central nervous system POMC is expressed in various cell types - inter alia in neurons of hypothalamus, arcuate nucleus, and the brain stem [28]. MC1R is primarily expressed in peripheral tissues, but also detectable in astrocytes, neurons of periaqueductal gray, and microvascular endothelial cells [29]–[31]. Its main physiological functions are the regulation of pigmentation and inflammatory response [32]. MC4R is expressed in brain primarily in astrocytes of cortex, thalamus, hypothalamus, and the brain stem [33]–[36]. The central anti-inflammatory actions of α-MSH are in part attributed to MC4R action [33], [37]. Furthermore, the melanocortin analog [Nle, D-Phe]-α-MSH demonstrated a MC4R-dependent protective effect in a diffuse rat TBI model [38]. The mRNA analysis in pericontusional tissue at 15 min, 6, 12, 24, and 48 h post TBI of the present study revealed that expression of POMC and MC4R is not influenced by mechanical brain lesion. This suggests that α-MSH levels are not endogenously upregulated upon injury via the precursor hormone and that MC4R expression is not regulated to modulate posttraumatic melanocortin-induced effects. In contrast, MC1R mRNA expression was about 3-fold increased at 12 h and remained elevated at 24 h and 48 h post injury. These results could indicate an increased interaction of melanocortins with MC1R following TBI, which is known for its high receptor affinity and association to regulation of inflammatory as well as immunomodulatory functions [39].

Exogenous application of the full-length amino-acid neuropeptide of α-MSH mediated neuroprotection and reduced tissue damage in experimental cerebral lesions like ischemic brain damage in models of focal and global cerebral ischemia or after spinal cord injury [25], [40]–[42]. Unfortunately, short half-life and skin pigmentation limit the use of α-MSH in clinical trials and initiated a quest for related peptide fragments and specific melanocortin receptor agonists [43]. The present study focused on KPV, the C-terminal tripeptide α-MSH(11–13). α-MSH(11–13), applied as single intraperitoneal injection 30 min after CCI, demonstrated an obvious reduction in neuronal apoptosis and brain lesion at 24 h after insult without melanotropic side effects of the complete functional protein. The secondary lesion volume was about 24% smaller in the tripeptide-injected group compared to vehicle, whereas the neurological outcome did not differ between groups. Possibly in the present study the brain trauma model was too mild to induce treatable motor dysfunctions. In addition, the chosen neurological outcome test was possibly not sensitive enough to reveal changes in general behavior or alertness between the investigated groups.

In fact, the data indicate a reduced secondary brain damage in α-MSH(11–13)- treated mice, which is caused at least in part by a reduction of apoptotic cell death. Following experimental TBI caspase-3 activation is one major modulating factor of apoptotic cell death [44], [45]. Therefore, in the present study immune-double staining for cleaved caspase-3 and NeuN was applied to investigate the influence of α-MSH(11–13) on neuronal apoptosis. Treatment with α-MSH(11–13) reduced considerably the number of apoptotic neurons (α-MSH(11–13) compared to vehicle: 26±10.3 to 54.8±19.4 cells/ROI). The specific pathways by which α-MSH(11–13) mediates its anti-apoptotic effects are part of ongoing discussions. In cell culture models induction of MC4R signaling prevented apoptosis e.g. via activation of extracellular signal-regulated kinase 1 and 2 (ERK1/2), inhibition of caspase-3 cleavage or modulation of Bax/Bcl-2 (Bcl-2 associated x protein/B-cell lymphoma 2) [33], [46]. These results are in line with anti-apoptotic effects of MC4R ligands observed in models of cerebral ischemia [14], [47], [48]. Although, in the present study MC4R was not upregulated after TBI, the regular concentration of these receptors might have been sufficient to successfully mediate the α-MSH(11–13) triggered anti-apoptotic effects. Up to now no evidence is provided that MC1R induces an anti-apoptotic effect via interaction with the α-MSH(11–13) tripeptide. Therefore, it is possible that the neuroprotective effect of α-MSH(11–13) after TBI might be in part caused by MC4R-mediated activation of anti-apoptotic pathways.

Secondary lesion expansion after TBI is also a result of cerebral inflammation [44], [49]. Following TBI infiltration of neutrophils, induction of nitric oxide synthase, release of free oxygen radicals and pro-inflammatory microglial cytokines contribute to the post-traumatic inflammatory response [50]–[52]. Accordingly, experimental and clinical trials seek for efficient anti-inflammatory therapies in the acute phase to reduce brain lesion [16], [53], [54]. One of these potential neuroprotective anti-inflammatory drugs is the investigated α-MSH(11–13). In order to provide evidence for the proposed influence of α-MSH(11–13) on inflammation as an underlying mechanism of protection, mRNA expression of inflammatory marker genes (TNF-α and IL-1β) were quantified in the present study, but failed to demonstrate differences between treatment and vehicle group. These results were unexpected, as the tripeptide α-MSH(11–13) is known to reduce expression of inflammatory marker genes like TNF-α, IL-6 and nitric oxide in vitro and to suppress lipopolysaccharide-induced NF-κB activation in vitro and in vivo [9], [55], [56]. In contrast, α-MSH(11–13) reduced IL-6 and IL-12 mRNA expression in a study investigating intestinal inflammation, while interferon-γ and IL-1β expression showed only a trend to decrease [57]. However, it could be hypothesized that the lack of reduction of cytokine expression in the present study is owed to the administration time 30 min post injury, because the reported lipopolysaccharide-induced NF-κB activation in a murine cerebral inflammation model was investigated after pretreatment with the tripeptide [56].

To specify differences in activation pattern of cerebral inflammation Iba-1 positive cells were quantified as marker for microglia activation. Microglia react rapidly within 30 min after injury and most of the surrounding cells of the cellular first line of defense migrate towards the area of injury within 1 to 3 h after insult [58]. The processes can persist for weeks or even years after e.g. human TBI, that reflects the dynamic, prolonged disorder in microglial activation after stoke or TBI [59]–[62]. Depending on the injury model microglial response may be influenced by injury severity and peaks around day 4 to 7 post CNS trauma [63]–[65]. Of particular note in the present study the microglia activation was investigated in a very early phase post injury. There was no difference in the number of activated microglia between groups 24 h after CCI, which matches with the cytokine expression data. For further analysis the grade of microglia activation was assessed by counting of their branches. Microglia activity was remarkably reduced in the tripeptide injected group, supporting an anti-inflammatory effect of α-MSH(11–13) as rather an immunomodulatory than immunosuppressive agent. In conclusion, there is evidence for a dual, anti-inflammatory and anti-apoptotic effect of α-MSH(11–13) as underlying mechanism for its neuroprotective property in the early phase after TBI.

In general the transferability of the study results to the clinical setting is limited by investigating mouse species with a single administration of a tripeptide [66]. Indeed, according to the criteria of good experimental practice drugs have to be tested in different animal models and conditions. The presented study represents a primary examination of α-MSH(11–13) in context of traumatic brain injury using a highly standardized CCI model.

### Conclusion

A single administration of the tripeptide α-MSH(11–13) limited secondary brain lesion expansion following TBI. The effect may be attributed to the known anti-inflammatory activity but furthermore an anti-apoptotic quality of the tripeptide and render α-MSH(11–13) a promising treatment option for therapeutic intervention after TBI.
